# Coloring the Mu transpososome

**DOI:** 10.1186/1471-2105-7-435

**Published:** 2006-10-05

**Authors:** Isabel K Darcy, Jeff Chang, Nathan Druivenga, Colin McKinney, Ram K Medikonduri, Stacy Mills, Junalyn Navarra-Madsen, Arun Ponnusamy, Jesse Sweet, Travis Thompson

**Affiliations:** 1 Mathematics Department, University of Iowa, Iowa City, IA 52242, USA; 2 Mathematics Department, University of Texas at Austin, Austin, TX 78712, USA; 3 Mathematics Department, Indiana University, Bloomington, IN 47405, USA; 4 Mathematics Department, Florida State University, Tallahassee, FL 32306, USA; 5 Mathematics Department, Texas Woman's University, Denton, TX 76204, USA; 6 Credit Suisse First, Boston, MA 02110, USA

## Abstract

**Background:**

Tangle analysis has been applied successfully to study proteins which bind two segments of DNA and can knot and link circular DNA. We show how tangle analysis can be extended to model any stable protein-DNA complex.

**Results:**

We discuss a computational method for finding the topological conformation of DNA bound within a protein complex. We use an elementary invariant from knot theory called colorability to encode and search for possible DNA conformations. We apply this method to analyze the experimental results of Pathania, Jayaram, and Harshey (Cell 2002). We show that the only topological DNA conformation bound by Mu transposase which is biologically likely is the five crossing solution found by Pathania *et al *(although other possibilities are discussed).

**Conclusion:**

Our algorithm can be used to analyze the results of the experimental technique described in Pathania *et al *in order to determine the topological conformation of DNA bound within a stable protein-DNA complex.

## Background

**Figure 1:**
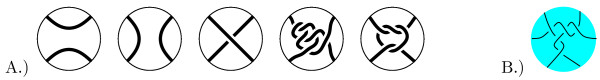
A.) Some 2-string tangles. B.) a 3-string tangle.

Tangles have many applications in modeling protein-DNA binding [1-5]. An *n-string tangle *consists of *n *strings properly embedded in a 3-dimensional (3D) ball. Some examples of 2-string tangles and a 3-string tangle are shown in Fig. 1. A protein complex bound to *n *segments of DNA can be modeled by an *n*-string tangle. The protein complex is modeled by the 3D ball while the *n *DNA segments can be thought of as *n *strings properly embedded in a protein ball (note each string represents one segment of double-stranded DNA). This is an extremely simple model of protein-DNA binding. A 3D ball does not accurately represent the shape of a protein complex, and DNA sometimes winds around a protein complex as opposed to being embedded within the protein complex. However, much information can be gained from this simple model.

When modeling protein-DNA reactions, it is helpful to know how to draw the DNA segments bound by the protein. For example, does the DNA molecule cross itself within the protein complex or does it bend sharply? Tangle analysis can be used to determine the topological shape of the DNA segments bound by a protein complex. Tangle analysis does not determine the exact geometry and hence cannot determine the sharpness of DNA bending, but it can determine the overall topology. This can be used to infer which DNA sequences are likely to be close to each other within the protein-DNA complex [5] as well as other information useful for modeling protein-DNA reactions.

The focus of this paper is two-fold: (1) we describe a computational method for solving *n*-string tangle equations for small crossing solutions; (2) we apply this method to analyze the topology of the DNA bound in the Mu transpososome.

Although our current C++ program is specific for analyzing the results of [5], we would be happy to make any necessary modifications for solving any other system of tangle equations for small crossing solutions, especially those modeling experimental data. The source code is also available upon request.

The Mu transpososome is involved in DNA transposition. *DNA transposition *is the process by which a piece of DNA can change its location within a genome. The Mu transposition pathway involves the formation of a series of protein-DNA complexes (for more biology background, see [5,6]). The *Mu transpososome *refers to the Mu transposase protein complex (Mu) and the three DNA segments bound by this protein complex. Since three DNA segments are bound by Mu, the Mu transpososome can be modeled by a 3-string tangle. An experimental technique called *difference topology *[7,5,10] combined with tangle analysis was used in [5] to determine that some of the Mu-DNA complexes can be modeled by the five crossing 3-string tangle shown in Fig. 1B. There are an infinite number of tangles that mathematically satisfy these experimental results (Darcy IK, Luecke J, Vazquez M: A tangle analysis of the Mu transpososome protein complex which binds three DNA segments, manuscript in preparation). These other conformations are very complicated and hence biologically unlikely to model the Mu transpososome, but they leave open the possibility that there are other biologically relevant models.

We describe a computational algorithm we have implemented which solves for biologically relevant topological conformations of DNA bound within the Mu transpososome using experimental results from [5]. For the purposes of this paper, we will consider a solution to be biologically relevant if it has a 2-dimensional projection with at most eight crossings. Observe that the solution found in [5] has five crossings (Fig. 1B). Although we briefly describe in the **Discussion and Conclusions **section why we believe the Mu transpososome contains at most eight crossings, our main reason for choosing to limit solutions up to eight crossings is computational time. Currently our C++ algorithm takes two days on a Linux computer with AMD Opteron Processor (2.2 GHz cpu) to find solutions up through eight crossings. The speed of the algorithm can be significantly improved by, for example, parallelizing the algorithm and running it on a cluster. Hence the algorithm can be improved to find solutions with around ten crossings. But as the number of tangles grows exponentially with crossing number, it is unlikely that this method can be used to find solutions with more than fifteen crossings due to computation time.

The experimental technique used in [5] can be applied to any protein complex which stably binds two or more segments of DNA (see **Discussion and Conclusions **for limitations) in order to determine the topological conformation of the DNA bound by the protein complex. The results of such experiments can be analyzed using a modification of the software we developed for analyzing the Mu experiments. In other words, this software can be modified to solve any system of *n*-string tangle equations for solutions containing up to around ten crossings, including those modeling difference topology experiments applied to a protein complex that stably binds any number of segments of DNA.

### An example of a tangle equation

**Figure 2:**
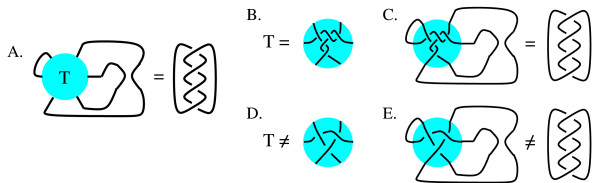
**A.) **An example of a tangle equation. **B.) **A solution to the tangle equation in (A). **C.) **The tangle equation from (A) where the tangle in (B) has been substituted for the tangle unknown **T **showing that the tangle in (B) is a solution to the tangle equation in (A). **D.) **A tangle which is not a solution to the tangle equation in (A). **E.) **When we plug the tangle in (D) into the equation in (A), we see that the three crossing tangle cannot result in a four crossing link for this equation. Hence this three crossing tangle is not a solution to the tangle equation in (A).

A description of the tangle equations modeling the difference topology experiments in [5] is given in [5] without the use of mathematical notation. Since we use mathematical notation, we start with an example of a tangle equation. Fig. 2A is a tangle equation with one unknown, the tangle **T**. A solution to this equation is a tangle **T **such that the conformation of the strands inside **T **combined with the conformation of the strands outside **T **must equal the four crossing link on the right-side of the equation in Fig. 2A. The tangle in Fig. 2B is a solution as shown in Fig. 2C. The three crossing tangle in Fig. 2D is not a solution to the tangle equation in Fig. 2A as shown in Fig. 2E.

### Cre recombinase

**Figure 3:**
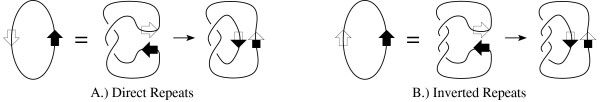
Cre recombination. A.) If the Cre binding sites are directly repeated, then Cre recombination results in a link. B.) If the Cre binding sites are inversely repeated, then Cre recombination can knot circular DNA.

Cre recombinase was used to obtain the system of tangle equations in [5] and hence we give some background information on Cre. Cre is a site-specific recombinase that will bind to 34 base pair DNA sequences called loxP. When Cre binds two copies of this sequence, it breaks both sequences and switches the ends before rejoining the DNA as shown in Fig. 3. If Cre acts on a circular DNA molecule containing Cre binding sites which are directly repeated as in Fig. 3A, then the resulting product is a two component link. If the circular DNA molecule contains inversely repeated Cre binding sites as illustrated Fig. 3B, then the product is a one component knot.

### Difference topology and tangle modeling

**Figure 4:**
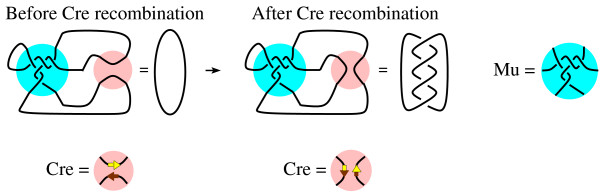
Difference topology experiment. Mu represented by the cyan colored ball is shown bound to five DNA crossings. Cre is represented by the smaller pink ball. Before Cre recombination, the DNA is circular and unknotted. Cre recombination changes the DNA configuration outside of the Mu transpososome. Since four of the five crossings bound by Mu are trapped by Cre recombination, the DNA product configuration equals a four crossing link.

We will now describe some of the difference topology experiments as well as the tangle model from [5]. The idea behind the experimental technique of difference topology is to use a protein such as Cre recombinase to trap crossings bound by the protein under study (in this case, Mu). This is illustrated in Fig. 4 where Mu is represented by the cyan colored ball. To show how a difference topology experiment works, we will assume the DNA conformation bound by Mu corresponds to the five crossing 3-string tangle in Fig. 1B based upon the results of [5]. In this technique, circular DNA is first incubated with the proteins involved in Mu transposition. The Mu complex binds DNA, possibly trapping DNA crossings within the protein complex. A second protein whose mechanism is well understood is added to the reaction (in this case, Cre, represented by smaller pink ball). This second protein, Cre, cuts the DNA and changes the circular DNA topology before resealing the breaks, resulting in knotted or linked DNA. The crossings bound by the first protein, Mu, will affect the product topology. In Fig. 4, four of the five crossings bound by Mu are trapped by the action of Cre, resulting in a four crossing link. Hence, one can gain information about the DNA conformation trapped by the first protein, Mu, by determining the knot/link type of the DNA knots/links produced by the second protein, Cre.

**Figure 5:**
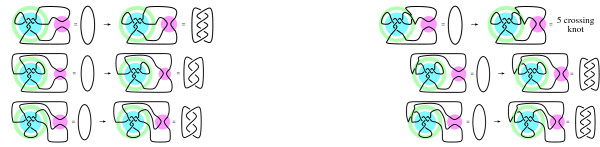
Tangle model from [5]. Mu is shown binding five DNA crossings (cyan ball). Cre recombination (pink balls) results in knotted and linked products. The topology of the knotted/linked products is dependent upon the location of the Cre binding sites and the DNA topology within the Mu transpososome.

Note that in the substrate configuration, three loops emanate from the Mu transpososome. The two binding sites for Cre can be placed in two of the three loops. By choosing on which pair of loops to place the Cre binding sites, the location of Cre action can be controlled. Six different substrates were constructed in [5] by varying the relative positions (choice of loop pairs) of the Cre sites as well as their relative orientations (direct versus inverted repeats). Models proposed in [5] of these six reactions are illustrated in Fig. 5. The cyan colored ball represents the DNA bound by Mu transposase while the pink colored ball represents the DNA bound by Cre.

Observe that in Fig. 4 (bottom) the yellow and brown arrow heads in the Cre complex point in opposite directions. Based on the crystal structure of Cre complexed with DNA [11], it was assumed in [5] that the two Cre binding sites must be in anti-parallel orientation with respect to each other within the Cre-DNA complex. Note that for the configuration in Fig. 3, an even number of crossings between Cre binding sites are needed to achieve an anti-parallel orientation between the Cre binding when the Cre sites are directly repeated (Fig. 3A) while an odd number of crossings are necessary when the Cre binding sites are inversely repeated (Fig. 3B). In the Mu/Cre models in Fig. 5, sometimes an extra crossing not bound by either protein is needed for correct DNA orientation within the Cre protein complex, depending on the orientation of the Cre binding sites on the two loops. When comparing products where the Cre sites are placed on the same pair of loops but in different orientations, it was assumed that the extra crossing occurred with the higher crossing product. When this extra crossing exists, it is placed within the green annulus in our figures. Hence crossings within the green annulus, if any, represent crossings not trapped by either protein complex.

**Figure 6:**
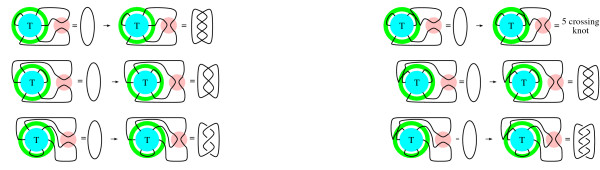
System of tangle equations corresponding to difference topology experiments in [5]. The tangle **T **(cyan ball) represents the unknown DNA conformation bound by Mu. In two of the experiments, the knot/link product was fully identified and hence we know that the crossings are all right-handed as shown in the bottom two tangle equations. In the remaining four experiments, only the crossing number of the knot/link was determined. There is only one three crossing knot and only one four crossing link up to mirror image. Hence, we know that for the three and four crossing products, the crossings are either all left-handed or all right-handed.

If we do not assume that the shape of DNA bound by Mu is the five crossing 3-string tangle from Fig. 1B, we can instead enclose the protein-bound DNA conformation into an unknown tangle, **T**. The system of tangle equations corresponding to these experiments is shown in Fig. 6 where the tangle **T **represents the unknown DNA conformation bound by Mu. When the Cre sites are directly repeated, the products are four crossing links regardless of the location of the Cre binding sites. The chirality of the four crossing links was only determined in one of the three cases where the Cre binding sites are directly repeated. But as there is only one four crossing link up to mirror image, the crossings of the two unidentified four crossing link products are either all left-handed or all right-handed. When the Cre sites are inversely repeated, the products are three crossing knots in two cases and a five crossing knot in the third case. Since there is only one three crossing knot up to mirror image, the crossings of the unidentified three crossing knot are either all left-handed or all right-handed. In **Methods**, we will prove mathematically that the five crossing knot must also contain all left-handed or all right-handed crossings, but for now we will make no assumptions regarding this knot other than that it contains five crossings as experimentally determined.

#### Mathematical model

*Determining the topological conformation of DNA bound by Mu is equivalent to solving the system of tangle equations in Fig*. 6* for the 3-string tangle ***T**. *A solution is a topological approximation, given as a 2-dimensional projection of a 3-dimensional conformation*.

An example of a 3-dimensional reconstruction using 2-dimensional tangle models is given in [3].

**Figure 7:**
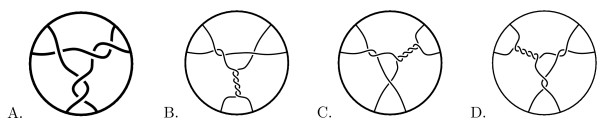
Three-branched supercoiled solutions to the tangle equations in Fig. 6.

In order to find the Fig. 1B solution, Pathania *et al *[5] assumed the protein-bound DNA is a 3-branched supercoiled structure like those in Fig. 7. Furthermore, since the substrate was negatively supercoiled unknotted DNA, Pathania *et al *[5] assumed that the crossings within each of the three branches is right-handed. Pathania *et al *[5] used the number of crossings in the knotted and linked DNA products to determine the number of crossings in each of the three branches in order to find the Fig. 1B solution which is repeated in Fig. 7A for convenience. Next, we illustrate their method for finding the number of crossings in each branch.

There exist four 3-branched supercoiled solutions to the tangle equations in Fig. 6. These solutions are shown in Fig. 7. They were obtained by solving a system of linear equations. For example, looking at the bottom left tangle equation in Fig. 6 in which the product is a right-handed three crossing knot, we observe that if the solution is a 3-branched supercoiled conformation with *x *crossings in one branch containing a Cre binding site and *y *crossings in the other branch containing the other Cre binding site, then *x *+ *y *= 3 (compare to bottom left tangle equation in Fig. 5). If we let *z *be the number of crossings in the third branch, then the top left equation involving an unidentified four crossing link in Fig. 6 corresponds to the linear equation *y *+ *z *= ± 4, while the equation involving the unidentified three crossing knot (middle left equation in Fig. 6) results in the equation *x *+ *z *= ± 3. If we solve the system of linear equations, *x *+ *y *= 3, *y *+* z *= ± 4, *x *+ *z *= ± 3, we obtain *x *= 1, *y *= 2, *z *= 2 (Fig. 7A), *x *= 2, *y *= 1, *z *= -5 (Fig. 7B), *x *= -2, *y *= 5, *z *= -1 (Fig. 7C), *x *= 5, *y *= -2, *z *= -2 (Fig. 7D).

Note that we are actually solving four different systems of linear equations (where each system has a unique 3-branched supercoiled solution) depending on whether the top left four crossing link is right- or left-handed (*y *+ *z *= ± 4) and whether the unidentified three crossing knot is right- or left-handed (*x *+ *z *= ± 3).

The solutions shown in Fig. 7B, 7C, 7D contain more crossings than the solution in Fig. 7A. Also, the solutions in Fig. 7B, 7C, 7D contain left-handed crossings. As the substrate DNA was negatively supercoiled, one would expect a 3-branched supercoiled structure to contain right-handed twists, not left-handed twists. Hence the Fig. 7A solution [5] is biologically more likely than the other 3-branched supercoiled solutions (Also, the solutions in Fig. 7B, 7C, do not satisfy additional experiments in [5] not described here).

The solutions in Fig. 7 are the only solutions if one considers only 3-branched supercoiled DNA conformations, but the question remains whether there are any other biologically relevant solutions if we do not assume a 3-branched supercoiled structure. In the next section, we describe colorability, the tangle invariant which we use to search for solutions for **T **where the only restriction placed on **T **is that it has eight or fewer crossings. However, a thorough understanding of this invariant is not necessary to understand the main idea behind the algorithm discussed in **Results**.

### The coloring invariants

**Figure 8:**
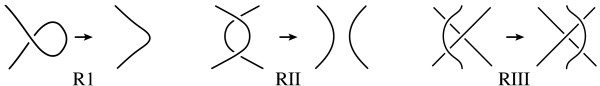
Reidemeister moves.

A *diagram*, *D*(**T**) of a knot, link, or tangle **T **is a projection of **T **into ℝ^2^, the 2-dimensional plane, where only double points are allowed at a crossing (two points are superimposed when strands cross), and gaps are used to indicate which part of the knot crosses under. Two diagrams correspond to the same 3D knot/link/tangle if one diagram can be converted to the other diagram via a sequence of Reidemeister moves-RI, RII, and RIII (Fig. 8).

**Figure 9:**
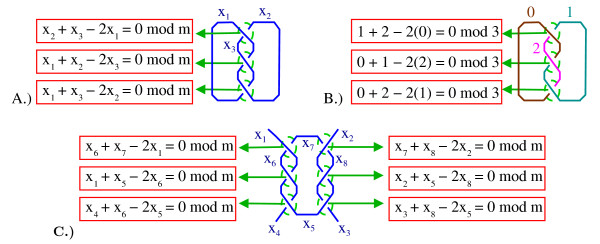
A.) Coloring a knot. The three arcs are labeled *x*_1_, *x*_2_, *x*_3_. A coloring of this knot diagram must satisfy the three equations corresponding to the three crossings. B.) A 3-coloring of this knot. C.) Coloring a 2-string tangle. The eight arcs are labeled *x*_1_, *x*_2_, ..., *x*_8_. The six crossings result in six equations.

An *m-coloring *of a diagram *D*(**T**) is a function *C *: {*arcs of D *(**T**)} ↦ ℤ
 MathType@MTEF@5@5@+=feaafiart1ev1aaatCvAUfKttLearuWrP9MDH5MBPbIqV92AaeXatLxBI9gBaebbnrfifHhDYfgasaacH8akY=wiFfYdH8Gipec8Eeeu0xXdbba9frFj0=OqFfea0dXdd9vqai=hGuQ8kuc9pgc9s8qqaq=dirpe0xb9q8qiLsFr0=vr0=vr0dc8meaabaqaciaacaGaaeqabaqabeGadaaakeaatuuDJXwAK1uy0HMmaeHbfv3ySLgzG0uy0HgiuD3BaGabaiab=rsiAbaa@3772@_*m *_where the elements of ℤ
 MathType@MTEF@5@5@+=feaafiart1ev1aaatCvAUfKttLearuWrP9MDH5MBPbIqV92AaeXatLxBI9gBaebbnrfifHhDYfgasaacH8akY=wiFfYdH8Gipec8Eeeu0xXdbba9frFj0=OqFfea0dXdd9vqai=hGuQ8kuc9pgc9s8qqaq=dirpe0xb9q8qiLsFr0=vr0=vr0dc8meaabaqaciaacaGaaeqabaqabeGadaaakeaatuuDJXwAK1uy0HMmaeHbfv3ySLgzG0uy0HgiuD3BaGabaiab=rsiAbaa@3772@_*m *_= {0,1, ..., m-1} will be called *colors*, and such that at each crossing the relation *y *+ *z *- 2*x *= 0 mod to holds, where *x *is the color assigned to the overarc and *y *and *z *are the colors of the two underarcs. See Fig. 9. A coloring is *trivial *if the coloring function is the constant map, i.e., all the arcs are assigned the same value or "color". A knot or link, **K **is said to be *in-colorable *if there exists a non-trivial *m*-coloring of *D*(**K**). This is a knot/link invariant in that if one diagram of the knot/link **K **is *m *colorable then all diagrams corresponding to **K **are *m*-colorable [12]. For an elementary introduction to coloring knots see [13]. We will more thoroughly define how coloring relates to tangles below [14,15].

A *coloring matrix *of a knot/link/tangle diagram, **T**, is any matrix, **M**_**T**_, which is row equivalent to a coefficient matrix corresponding to the coloring equations. For example, the 6 × 8 matrix in Eqn. (1) is a coloring matrix corresponding to the tangle diagram in Fig. 9C. Each row corresponds to one of the six crossings in the tangle diagram while each column represents one of the eight arcs, *x*_5_, *x*_6_, *x*_7_, *x*_8_, *x*_1_, *x*_2_, *x*_3_, *x*_4 _in the tangle diagram.

(0110−20001−2001000−2100000100110−200100−20100−20010010)×(x5x6x7x8x1x2x3x4)=(00000000) mod m     (1)
 MathType@MTEF@5@5@+=feaafiart1ev1aaatCvAUfKttLearuWrP9MDH5MBPbIqV92AaeXatLxBI9gBaebbnrfifHhDYfgasaacH8akY=wiFfYdH8Gipec8Eeeu0xXdbba9frFj0=OqFfea0dXdd9vqai=hGuQ8kuc9pgc9s8qqaq=dirpe0xb9q8qiLsFr0=vr0=vr0dc8meaabaqaciaacaGaaeqabaqabeGadaaakeaadaqadaqaauaabeqagGaaaaaaaeaacqaIWaamaeaacqaIXaqmaeaacqaIXaqmaeaacqaIWaamaeaacqGHsislcqaIYaGmaeaacqaIWaamaeaacqaIWaamaeaacqaIWaamaeaacqaIXaqmaeaacqGHsislcqaIYaGmaeaacqaIWaamaeaacqaIWaamaeaacqaIXaqmaeaacqaIWaamaeaacqaIWaamaeaacqaIWaamaeaacqGHsislcqaIYaGmaeaacqaIXaqmaeaacqaIWaamaeaacqaIWaamaeaacqaIWaamaeaacqaIWaamaeaacqaIWaamaeaacqaIXaqmaeaacqaIWaamaeaacqaIWaamaeaacqaIXaqmaeaacqaIXaqmaeaacqaIWaamaeaacqGHsislcqaIYaGmaeaacqaIWaamaeaacqaIWaamaeaacqaIXaqmaeaacqaIWaamaeaacqaIWaamaeaacqGHsislcqaIYaGmaeaacqaIWaamaeaacqaIXaqmaeaacqaIWaamaeaacqaIWaamaeaacqGHsislcqaIYaGmaeaacqaIWaamaeaacqaIWaamaeaacqaIXaqmaeaacqaIWaamaeaacqaIWaamaeaacqaIXaqmaeaacqaIWaamaaaacaGLOaGaayzkaaGaey41aq7aaeWaaeaafaqabeacbaaaaaqaaiabdIha4naaBaaaleaacqaI1aqnaeqaaaGcbaGaemiEaG3aaSbaaSqaaiabiAda2aqabaaakeaacqWG4baEdaWgaaWcbaGaeG4naCdabeaaaOqaaiabdIha4naaBaaaleaacqaI4aaoaeqaaaGcbaGaemiEaG3aaSbaaSqaaiabigdaXaqabaaakeaacqWG4baEdaWgaaWcbaGaeGOmaidabeaaaOqaaiabdIha4naaBaaaleaacqaIZaWmaeqaaaGcbaGaemiEaG3aaSbaaSqaaiabisda0aqabaaaaaGccaGLOaGaayzkaaGaeyypa0ZaaeWaaeaafaqabeacbaaaaaqaaiabicdaWaqaaiabicdaWaqaaiabicdaWaqaaiabicdaWaqaaiabicdaWaqaaiabicdaWaqaaiabicdaWaqaaiabicdaWaaaaiaawIcacaGLPaaacqqGGaaiieGacqWFTbqBcqWFVbWBcqWFKbazcqqGGaaicqWGTbqBcaWLjaGaaCzcamaabmaabaGaeGymaedacaGLOaGaayzkaaaaaa@8EA2@

We will call the arcs which have one endpoint on the boundary of the tangle 3-ball *endpoint arcs*. The remaining arcs will be called *interior arcs*. Notice that we place the four columns corresponding to the endpoint arcs, *x*_1_, *x*_2_, *x*_3_, *x*_4_, as the four rightmost columns of the matrix **M**_**T **_. We can solve this system of equations by performing the following row operations: (1) exchange two rows (*row i *↔ *row j*); (2) add a multiple of one row to a different row (*row i *→ *row i *+ *t *· *row j*, *i *≠ *j*, *t *∈ ℤ
 MathType@MTEF@5@5@+=feaafiart1ev1aaatCvAUfKttLearuWrP9MDH5MBPbIqV92AaeXatLxBI9gBaebbnrfifHhDYfgasaacH8akY=wiFfYdH8Gipec8Eeeu0xXdbba9frFj0=OqFfea0dXdd9vqai=hGuQ8kuc9pgc9s8qqaq=dirpe0xb9q8qiLsFr0=vr0=vr0dc8meaabaqaciaacaGaaeqabaqabeGadaaakeaatuuDJXwAK1uy0HMmaeHbfv3ySLgzG0uy0HgiuD3BaGabaiab=rsiAbaa@3772@); (3) multiply a row by -1 (*row i *↔ -*row i*). Since we are working in ℤ
 MathType@MTEF@5@5@+=feaafiart1ev1aaatCvAUfKttLearuWrP9MDH5MBPbIqV92AaeXatLxBI9gBaebbnrfifHhDYfgasaacH8akY=wiFfYdH8Gipec8Eeeu0xXdbba9frFj0=OqFfea0dXdd9vqai=hGuQ8kuc9pgc9s8qqaq=dirpe0xb9q8qiLsFr0=vr0=vr0dc8meaabaqaciaacaGaaeqabaqabeGadaaakeaatuuDJXwAK1uy0HMmaeHbfv3ySLgzG0uy0HgiuD3BaGabaiab=rsiAbaa@3772@_*m *_where to is an arbitrary integer, scaling a row is not allowed.

The first non-zero term in a row is called a leading entry. A matrix is in echelon form if (1) rows consisting of only zero's occur below rows containing at least one non-zero term; (2) each entry below a leading entry is zero; (3) If *a*_*ih *_and *a*_*jk *_are leading entries and if *i *<*j*, then *h *<*k *(i.e., the leading entries move to the right as the rows descend). An echelon form, *EF*(**M**_**T**_) of the matrix in Eqn. (1) is

EF(MT)=(100−201000110−200000110−20000030−2−1000001−11−10000003−3)     (2)
 MathType@MTEF@5@5@+=feaafiart1ev1aaatCvAUfKttLearuWrP9MDH5MBPbIqV92AaeXatLxBI9gBaebbnrfifHhDYfgasaacH8akY=wiFfYdH8Gipec8Eeeu0xXdbba9frFj0=OqFfea0dXdd9vqai=hGuQ8kuc9pgc9s8qqaq=dirpe0xb9q8qiLsFr0=vr0=vr0dc8meaabaqaciaacaGaaeqabaqabeGadaaakeaacqWGfbqrcqWGgbGrcqGGOaakieqacqWFnbqtdaWgaaWcbaGae8hvaqfabeaakiabcMcaPiabg2da9maabmaabaqbaeqabyacaeaabaqaaiabigdaXaqaaiabicdaWaqaaiabicdaWaqaaiabgkHiTiabikdaYaqaaiabicdaWaqaaiabigdaXaqaaiabicdaWaqaaiabicdaWaqaaiabicdaWaqaaiabigdaXaqaaiabigdaXaqaaiabicdaWaqaaiabgkHiTiabikdaYaqaaiabicdaWaqaaiabicdaWaqaaiabicdaWaqaaiabicdaWaqaaiabicdaWaqaaiabigdaXaqaaiabigdaXaqaaiabicdaWaqaaiabgkHiTiabikdaYaqaaiabicdaWaqaaiabicdaWaqaaiabicdaWaqaaiabicdaWaqaaiabicdaWaqaaiabiodaZaqaaiabicdaWaqaaiabgkHiTiabikdaYaqaaiabgkHiTiabigdaXaqaaiabicdaWaqaaiabicdaWaqaaiabicdaWaqaaiabicdaWaqaaiabicdaWaqaaiabigdaXaqaaiabgkHiTiabigdaXaqaaiabigdaXaqaaiabgkHiTiabigdaXaqaaiabicdaWaqaaiabicdaWaqaaiabicdaWaqaaiabicdaWaqaaiabicdaWaqaaiabicdaWaqaaiabiodaZaqaaiabgkHiTiabiodaZaaaaiaawIcacaGLPaaacaWLjaGaaCzcamaabmaabaGaeGOmaidacaGLOaGaayzkaaaaaa@6DE8@

We define the standard echelon form of a matrix, *SF*(**M**), to be the echelon form in which each leading entry is positive and if *a*_*ij *_is a leading entry of the *i*th row, then 0 ≤ *a*_*rj *_≤ *a*_*ij *_-1, 1 ≤ *r *<*i*. The standard echelon form of a matrix is unique. Note that the matrix in Eqn. (2) is not in standard echelon form, but the lower right hand corner 2 × 4 submatrix is in standard echelon form (see also Eqn. (3)).

Let **M**_**l**_(**T**) be the lower right hand corner 2 × 4 submatrix of **M**_**T **_in standard echelon form. If the endpoints arcs' unknowns, *x*_1_, *x*_2_, *x*_3_, *x*_4 _correspond to the four rightmost columns, then **M**_**l**_(**T**) is a tangle invariant. It is a tangle invariant in that if you take two diagrams of the same tangle **T **and place the endpoint arcs in the same order in the last columns of their respective coloring matrices, then no matter how the interior arcs are labeled, **M**_**l**_(**T**) will be the same for both diagrams. In addition, the absolute value of the determinant of the upper left 4 × 4 submatrix, **d**_**u**_(**T**) = 3, is also an invariant.

Ml(T)=(1−11−1003−3),du(T)=3     (3)
 MathType@MTEF@5@5@+=feaafiart1ev1aaatCvAUfKttLearuWrP9MDH5MBPbIqV92AaeXatLxBI9gBamXvP5wqSXMqHnxAJn0BKvguHDwzZbqegyvzYrwyUfgarqqtubsr4rNCHbGeaGqiA8vkIkVAFgIELiFeLkFeLk=iY=Hhbbf9v8qqaqFr0xc9pk0xbba9q8WqFfeaY=biLkVcLq=JHqVepeea0=as0db9vqpepesP0xe9Fve9Fve9GapdbaqaaeGacaGaaiaabeqaamqadiabaaGcbaqbaeqabeGaaaqaaGWabiaa=1eacaWFSbGaeiikaGIaa8hvaiabcMcaPiabg2da9maabmaabaqbaeqabiabaaaabaGaeGymaedabaGaeyOeI0IaeGymaedabaGaeGymaedabaGaeyOeI0IaeGymaedabaGaeGimaadabaGaeGimaadabaGaeG4mamdabaGaeyOeI0IaeG4mamdaaaGaayjkaiaawMcaaiabcYcaSaqaaiaa=rgadaWgaaWcbaGaa8xDaaqabaGccqGGOaakcaWFubGaeiykaKIaeyypa0JaeG4mamdaaiaaxMaacaWLjaWaaeWaaeaacqaIZaWmaiaawIcacaGLPaaaaaa@5969@

In the above example, the tangle diagram **T **is a 2-string tangle with six crossings. Hence its coloring matrix is a 6 × (6 + 2) = 6 × 8 matrix, and we were interested in the 2 × 4 matrix **M**_**l**_(**T**) as well as the determinant of the upper left 4 × 4 matrix. In the general case, suppose **T **is a diagram of an arbitrary *n*-string tangle with a *k *× (*k *+ *n*) coloring matrix **M**_**T **_(listing the 2*n *endpoint arcs in the right-most columns of the matrix in a fixed order). Let **M**_**l**_(**T**) be the lower right-hand corner *n *× 2*n *submatrix of **M**_**T **_in standard echelon form, and let **d**_**u**_(**T**) be the absolute value of the determinant of the upper left (*k *- *n*) × (*k *- *n*) submatrix of **M**_**T **_. Both **M**_**l**_(**T**) and **d**_**u**_(**T**) are invariants of **T **[15]. Note that columns corresponding to the endpoint arcs must be the right-most columns of the coloring matrix, and these columns must be in a fixed order when calculating **M**_**l**_(**T**). We will always order the endpoint arcs in a clockwise manner starting with a northwest endpoint arc.

In order to calculate **M**_**l**_(**T**) where **T **is an *n*-string tangle, we must label 2*n *endpoint arcs with distinct variables. If a string consists of just one arc (i.e., a string does not pass under any other string including itself so that it projects to just one arc; hence both endpoints of this arc lie on the boundary of the 3D ball), we can doubly label the arc, labeling one half of this endpoint arc *x*_*i*_, the other half *x*_*j*_, and adding the equation *x*_*i *_- *x*_*j *_= 0. Normally an *n*-string tangle with *k *crossings will have a *k *× (*k *+ *n*) coloring matrix. But if any arcs are doubly labeled, then the coloring matrix will have more than *k *rows and (*k *+ *n*) columns.

## Results

We describe a computational algorithm we have implemented to solve the system of tangle equations in Fig. 6. The full description is given in **Methods**. The majority of the algorithms were written so that this program can easily be modified to solve any system of *n*-string tangle equations up to around 8–10 crossings, including those modeling difference topology experiments applied to a protein complex that stably binds any number of segments of DNA.

**Figure 10:**
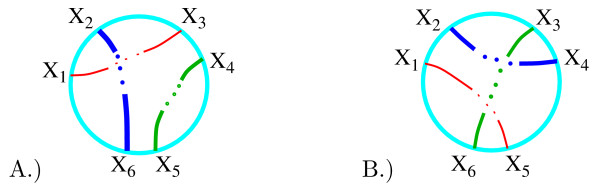
Possible parities.

We first determine how the strings enter and exit the tangle. The parity of a tangle refers to the order in which the strings enter and exit the 3D ball. A solution to the tangle equations in Fig. 6 can have one of two possible parities: the strings enter and exit the tangle as in Fig. 10A or as in Fig. 10B. This is easily determined by noting which of the equations in Fig. 6 involve a knot (one component) versus a two component link. For example, the string entering in at *x*_1 _cannot exit at *x*_2 _since the top left equation in Fig. 6 involves the one component unknot. As discussed in **Methods, **we also use 2-string tangle analysis to simplify the system of equations in Fig. 6.

A number of techniques have been used to encode knot diagrams for computational purposes [16,17]. As described in **Methods**, we use coloring matrices to encode tangle diagrams. We generate matrices which could correspond to tangle diagrams up through eight crossings. We check each matrix to determine if it has the correct coloring invariants to be a solution to the tangle equations in Fig. 6. As shown in table 1, this eliminates the majority of the generated matrices. Not all generated matrices correspond to a tangle. We use an algorithm similar to that described in [18] to remove all matrices which do not correspond to a tangle.

**Table 1:** Results.

#of Crossings	# of Matrices Generated	Parity Fig. 10A			Parity Fig. 10B		
		Col	Draw	Non-Equiv?	Col	Draw	Non-Equiv?
≥ 4	1,639	0	0	0	0	0	0
5	34,578	1	1	1	1	0	0
6	794,578	22	4	0	22	0	0
7	19,781,058	354	15	3	400	0	0
8	537,193,563	5019	106	6	5595	6	3

total (≥ 8)				10			3

Number of matrices with the correct coloring invariants (Col columns), corresponding to a drawable tangle (Draw columns), and which are potentially non-equivalent (Non-equiv columns). The first column refers to the number of crossings in the tangle diagram. The second column gives the number of matrices generated which could correspond to a tangle with a fixed crossing number. The results in the next three columns assume the parity in Fig. 10A while the results in the last three columns assume the parity in Fig. 10B. The columns labeled "Col" state the number of generated matrices which have the correct coloring invariants to satisfy the equations in Fig. 6. However, not all generated matrices correspond to a tangle. The columns labeled "Draw" give the number of matrices which correspond to a drawable tangle with the correct coloring invariants. The number of these matrices which may correspond to non-equivalent tangles is given in the columns labeled "Non-equiv?". Note, however, that the algorithm does not identify all equivalent tangles.

Recall that a tangle can be represented by a number of different diagrams related by Reidemeister moves. Unfortunately, there is no algorithm guaranteed to determine whether two tangle diagrams are equivalent. In fact, in order to simplify a diagram, it may be necessary to first increase the number of crossings in the diagram. Thus this software does not determine all tangle equivalences, but does reduce the output sufficiently to handle the remaining possibly equivalent tangles by hand. While generating matrices, we omit matrices where the corresponding diagram can be simplified by RI or RII moves (Fig.8). As discussed in **Methods**, we also perform some other simplifications which involve a combination of RI, RII, and RIII moves. As shown in table 1, this leaves us with 13 matrices that could correspond to tangles satisfying the system of equations in Fig. 6: ten with the parity shown in Fig. 10A and three with the parity shown in Fig. 10B.

We checked the remaining thirteen tangles corresponding to these matrices by hand. The ten tangles with Fig. 10A parity are all equivalent to the five crossing tangle found in [5] (Fig. 7A). The three tangles with Fig. 10B parity are all equivalent to one of the two eight crossing tangles in Fig. 7B, C. Recall that the two eight crossing solutions were not considered in [5] since the unknotted DNA substrate was negatively supercoiled and hence trapping left-handed crossings is biologically unlikely.

## Discussion

We have developed software to analyze the difference topology experiments in [5]. Pathania *et al *[5] needed to assume the basic shape of a 3-branched supercoiled structure (Fig. 7) in order to find the solution shown in Fig. 1B (= Fig. 7A). With our software, no assumptions regarding the DNA conformation bound by the protein complex are needed except for an upper bound on the number of crossings. This algorithm can also be modified to analyze any difference topology experiment regardless of the number of DNA segments bound by the protein complex (although there is a bound on the topological complexity of the protein-bound DNA as discussed below).

A tangle solution is a topological approximation given as a 2-dimensional projection of a 3-dimensional structure. It does not determine sharpness of DNA bending, but it does give an important starting point from which other modeling techniques may be applied. Limited information regarding the Mu-DNA conformation existed before [5]. Since then a partial structure based on scanning transmission electron microscopy (STEM) at cryo-temperatures has become available [19], but this involves only a portion of the protein complex and a change in the DNA sequences bound by Mu. Information regarding protein-bound DNA conformations can sometimes be obtained via crystallography, STEM, or FRET (fluorescence resonance energy transfer), but all these techniques are quite difficult and currently can only be applied to small protein-DNA complexes.

Recall that in the Mu tangle model from [5] (Figs. 5, 6), it is assumed that at most one crossing is trapped outside of the protein complexes (modeled within the green annulus). Since Mu and Cre bind to specific DNA sequences, the length of the DNA between the Mu binding sites and Cre binding sites can be controlled. The shortest length needed for the reaction to take place was determined in [5] in order to prevent trapping extraneous crossings. The difference topology experimental technique can also be applied to proteins that bind to arbitrary DNA sequences rather than specific DNA sequences, but the results would not be expected to be as clean (both in terms of experimental results as well as determining the appropriate tangle model). It was shown in [20] that if the length of DNA between binding sites is not properly controlled, then the number of protein-bound DNA crossings may be overestimated. But even if we are left with a topological approximation, it is still a significant improvement over having little or no information on how to draw the DNA in a protein-DNA complex.

We are not mathematically limited to equations resulting from Cre recombination. Any protein which can change DNA topology could potentially be used in a set of difference topology experiments to obtain a different system of tangle equations. For example topoisomerases change the topology of circular DNA by changing DNA crossings. It may be possible to obtain a more 3-dimensional model by averaging 2-dimensional projections of tangle solutions from two or more systems of tangle equations or tangle models [3,4]. Cre, however, may be the easiest to work with due to its sequence specificity and its simple mechanism.

### The software and its applicability to *n*-string tangle equations

This software consists of 4 steps:

1. Matrices which could correspond to coloring matrices of tangle diagrams are generated (see subsection **Tangle generation **in **Methods**)

2. The coloring invariants of each matrix are checked (subsection **Checking the coloring invariants **in **Methods**). Implementing this part of the software requires that we first mathematically simplify the system of tangle equations via 2-string tangle analysis (subsection **2-string tangle simplification **in **Methods**).

3. Not all the matrices generated in step 1 will correspond to a tangle diagram. Hence each generated matrix is checked to determine if it actually corresponds to a tangle diagram (subsection **Non-drawable matrices **in **Methods**).

4. Different matrices can correspond to the same tangle. Thus we remove some (but not all) of the redundant matrices (subsection **Equivalence moves **in **Methods**)

No modifications are needed for Steps 1 and 3 in order to apply this algorithm to a different system of *n*-string tangle equations. For step 2, additional invariants may be needed in addition to or in replacement of the coloring invariants. Additional subroutines may also be needed for step 4.

Although coloring is not that powerful of a knot invariant, it is a powerful tangle invariant. As our results show, it is the only invariant we need to check to determine if a tangle up through eight crossings is a solution to the equations in Fig. 6. However, there is no guarantee that this invariant will be sufficient for a different system of tangle equations. Hence we may need to check additional invariants. Fortunately, there are a number of other invariants as well as software available for calculating these other invariants which can be used when needed [17,21]. In particular we plan to add the homflypt polynomial knot invariant as an alternative option to the coloring invariant. The homflypt polynomial has been used in other algorithms requiring computational speed [22]. Knots with nine or fewer crossings are uniquely identified by their homflypt polynomial. Hence if the knotted products of the difference topology experiments contain fewer than ten crossings, then checking the homflypt polynomial is sufficient (i.e., the homflypt polynomial will completely determine if a tangle is a solution to a system of *n*-string tangle equations if the equations only involve knots with less than ten crossings). Even if we need to use different invariant(s), this does not affect any other part of the algorithm. In particular, we can still use coloring matrices to encode tangle diagrams.

Our software left us with only 13 different coloring matrices which could correspond to tangle solutions to the system of equations in Fig. 6. We could have added additional methods to determine if two tangle diagrams are equivalent to further reduce this output, but it was quicker to check these 13 matrices by hand. For a different system of equations, additional methods to determine tangle equivalence may be needed to reduce the output to a handful of matrices. We will add additional subroutines to decrease the number of redundant tangles as needed.

The modifications that may be needed are straightforward. In fact they have been used by others for a computationally much more complex problem, knot tabulation [16]. The techniques we use are very similar to those used to tabulate knots up through 16 crossings. The main difference between knot tabulation and our algorithm is that in tabulating knots, every knot diagram must be fully identified and all redundancies eliminated. In our algorithm, we discard diagrams that do not satisfy our equations, and hence only need to analyze a very small fraction of diagrams compared to the number of diagrams analyzed in knot tabulation. Also, since we focus on only a few systems of equations at a time, we can analyze by hand some redundancies among our tangle solutions. Hence we don't need to check nearly as many invariants or computationally determine as many tangle equivalences as in knot tabulation where millions of knots have been identified [23]. Thus our algorithm is computationally much simpler than that required for knot tabulation.

Unfortunately, we cannot give a mathematical estimate regarding the number of solutions or the number of redundancies for an arbitrary system of tangle equations. In most cases, any modifications needed to reduce the number of repeated solutions will take at most a few days to implement. However, if the system of tangle equations is under-determined so that it has many small crossing solutions, then determining redundancies computationally will become much more important. An example of an under-determined system would be one modeling a partial set of difference topology experiments. In [5], Cre binding sites, in both inverted and direct orientations, were placed on each pair of the three loops emanating from the Mu transpososome. Hence six different substrates were constructed. If a protein binds, for example, four segments of DNA, then four loops will emanate from the protein-DNA complex. If Cre binding sites are placed on each pair of these four loops in both inverted and direct orientation, twelve substrates would be needed. In general if a protein-complex binds *n *segments of DNA, one would need to contract *n*(*n *- 1) different substrates if Cre binding sites are placed on each pair of loops in both orientations. An under-determined system would result if Cre binding sites are not placed on each pair of loops. We will eventually be able to solve under-determined systems for small crossing solutions as this problem is still much simpler than knot tabulation, but we expect this will take longer to implement.

### Other mathematical methods

There are many mathematical techniques (for example [1,24-36]) as well as software [37,38] for solving 2-string tangle equations. Hence many (but not all) biologically relevant 2-string tangle equations can be completely solved. Similar mathematics does not yet exist for solving *n*-string tangle equations for *n *> 2. Some work has been done on 3-string tangles [39] and solving 3-string tangles equations involving the class of 3-string tangles called 3-braids [40]. There is also some work on classifying *n*-string tangles (for example, [41]). Also techniques in 3-manifold theory can be applied to solve *n*-string tangle equations for small crossing solutions [42], (Darcy IK, Luecke J, Vazquez M: A tangle analysis of the Mu transpososome protein complex which binds three DNA segments, manuscript in preparation). However, at the moment, there are no mathematical methods for solving the system of 3-string tangle equations in Fig. 6 or for most systems of *n*-string tangle equations.

### Computational limitations

Currently this C++ algorithm takes about two days on a Linux computer with AMD Opteron Processor (2.2 GHz cpu) to find solutions through eight crossings. However, the efficiency of the algorithm can be significantly improved by parallelizing it and running it on a cluster. Hence it should be possible to find solutions up to about ten crossings. As the number of tangles grows exponentially with crossing number, this algorithm can not be used to find high crossing solutions. Knots have only been tabulated up through sixteen crossings. Although our algorithm is computationally much simpler than knot tabulation, there are more tangles with *k *crossings than there are knots with *k *crossings. Hence we do not expect to be able to get much past ten crossings with a reasonable computation time.

Despite this computational limitation, we believe this algorithm is applicable to a wide array of protein-DNA complexes. The length of DNA bound by the protein limits the bound DNA's topological complexity. For example, the three DNA segments bound within the Mu transpososome are 50, 175 and 190 base pairs. However, we do not know of a theoretical tipper bound on the topological complexity of protein-bound DNA.

We believe eight crossings is a reasonable limit for the Mu transpososome. In addition to limits imposed by the lengths of the three protein-bound DNA sequences, the existence of a five crossing solution implies that a much more complicated solution with eight or more crossing is less likely. However, we have no proof that this is the case.

## Conclusion

The computational algorithm described in this paper can be modified to solve any system of *n*-string tangle equations for small crossing tangle solutions. A long-term goal is to create software accessible to those without a background in knot theory. Eventually this software will be able to draw the tangle solutions. Some additional work is needed to handle under-determined systems of tangle equations as discussed above. But in the meantime if the system is not under-determined, we can readily modify this algorithm to solve any specified system of tangle equations up to around ten crossings; hence an experimentalist need not wait for the final version of this software before performing difference topology experiments.

## Methods

### Tangle generation

**Figure 11:**
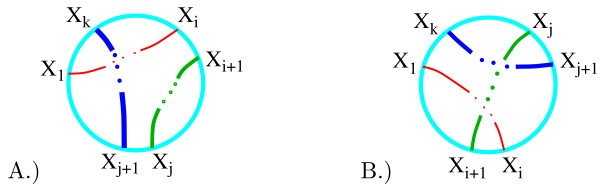
Possible parities. Note that the endpoint arcs are neither labeled consecutively nor in a clockwise fashion for tangle generation.

We use the coloring matrix of a tangle diagram to encode its shape. Recall that a solution to the tangle equations in Fig. 6 can have one of two possible parities: the strings enter and exit the tangle as in Fig. 11A or as in Fig. 11B. For tangle generation, we will not place the columns corresponding to the endpoint arcs in the rightmost columns. This simplifies the matrix generation as well as determining if a matrix corresponds to a drawable tangle or if two matrices correspond to the same tangle. In order to calculate the coloring invariants, we will later move the columns corresponding to the endpoint arcs to the rightmost columns. The red string which begins with the endpoint arc labeled *x*_1 _and ends with the endpoint arc *x*_*i *_will be called string 1. The green string which begins with the endpoint arc labeled *x*_*i*+*i *_and ends with the endpoint arc *x*_*j *_will be called string 2 while the remaining blue string will be called string 3.

**Figure 12:**
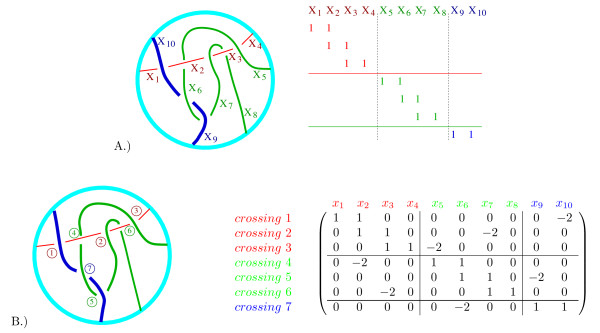
A.) Example: labeling arcs. The arcs correspond to columns in the coloring matrix. The rows of the coloring matrix are not determined until the crossings are labeled, but are included in the above figure for illustrative purposes. The matrix is partitioned into blocks in order to emphasize the correlation between the placement of 1's and the number of arcs in each string. Observe 1's only occur in the diagonal blocks in the pattern shown. B.) Example: labeling crossings.

We first consecutively label the arcs of red string 1 beginning with *x*_1 _as illustrated with the example in Fig. 12A. The red string is broken into four arcs with the arcs consecutively labeled *x*_1_, *x*_2_, *x*_3_, *x*_4_. We then label the arcs of the green second string starting from the first endpoint arc clockwise from the red endpoint arc *x*_4_. String 2 is broken into four arcs which are consecutively labeled *x*_5_, *x*_6_, *x*_7_, *x*_8_. We then label the arcs of string 3, *x*_9_, *x*_10_, starting from the first endpoint arc clockwise from the last labeled arc of string 2. Recall that the arcs correspond to columns in the coloring matrix (Fig. 12A). Hence for tangle generation, we have chosen a particular ordering of the columns by ordering the arcs.

Recall that the coloring equations (which correspond to rows in the coloring matrix) are determined by the crossings in the tangle diagram. Hence we determine the ordering of the rows by labeling the crossings. Beginning with string 1, we consecutively number the under-crossings (Fig. 12B). Hence for string 1, crossing number *i *occurs between string 1 arcs *x*_*i *_and *x*_*i*+*i*_. For string 2, crossing number *j *occurs between string 2 arcs *x*_*j*+1 _and *x*_*j*+2 _while for string 3, crossing number *k *occurs between string 3 arcs *x*_*k*+2 _and *x*_*k*+3_. This determines the placement of the two 1's in each row (Fig. 12B). To generate matrices that could correspond to a coloring matrix, we can now place one -2 in each row in all possible combinations.

Not all matrices that could correspond to a 3-string tangle are generated (see below). Not all generated matrices correspond to a tangle (see section on **Non-drawable matrices**). Many different matrices correspond to the same tangle (see below and section on **Equivalence moves**).

#### Matrices not generated

The algorithm under discussion does not generate all matrices which could correspond to a tangle. A tangle diagram can contain an extraneous crossing manifested by the looping of a string over itself. If the loop does not pass under any string, this results in the equation *x*_*i *_- *x*_*i*+1 _= 0.  This is more general than an RI move (Fig. 8) as there could be strings passing under this loop. In any case this tangle diagram can be simplified, and hence we do not need to generate the matrix corresponding to this diagram. Since all matrices generated have two "l"s and one "-2" in each row, none of the matrices generated will correspond to a tangle containing such an extraneous crossing.

Another case that is not generated is the presence of a string not crossing under any arcs, and hence consisting of just one arc doubly labeled *x*_*i *_and *x*_*i*+1 _. This case results in the equation, *x*_*i *_- *x*_*i*+1 _= 0. We could easily generate this, but the system of tangle equations in Fig. 6 rules out such tangles as possible solutions.

**Figure 13:**
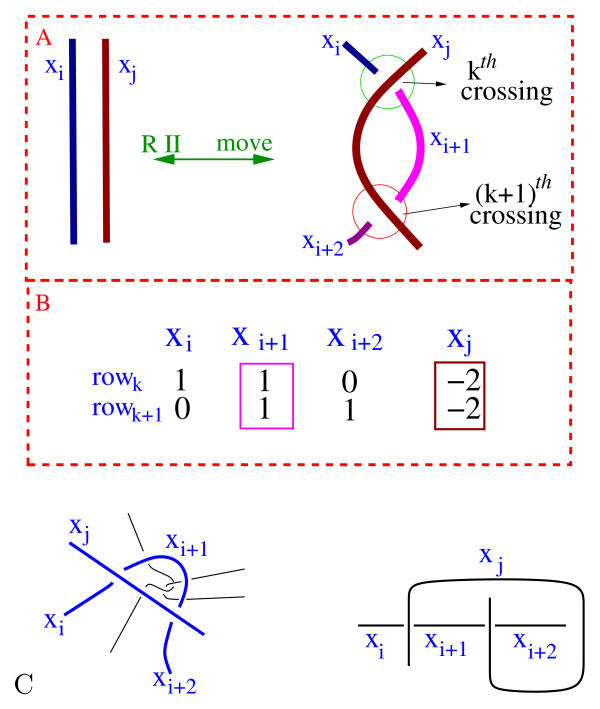
A.) An RII move. B.) Matrix corresponding to RII move. C.) Tangles which would also contain the submatrix in Fig. 13B.

The algorithm also does not generate matrices that correspond to tangles containing crossings which can be removed by an RII move. These matrices contain -2's in the same column in two consecutive rows where the rows correspond to the same string. See Fig. 13. By not generating matrices containing the submatrix in Fig. 13B, we do not generate any tangle diagrams which can be simplified by an RII move (Fig. 13A). This also eliminates other tangles whose coloring matrix also contains this submatrix. This includes tangle diagrams containing a generalization of an RII move where strings are allowed to pass under the strings which would otherwise correspond to an RII move (Fig. 13C, left-side) as well as tangles containing diagrams like that on the right-side of Fig. 13C. All of these tangle diagrams can be simplified. This is one advantage of using coloring matrices to generate tangles: we easily remove a number of matrices that correspond to tangle diagrams where the number of crossings can be reduced.

The next part of this software checks the coloring invariant as this removes the majority of the matrices from consideration. However, for readability, we will discuss the drawability algorithm first.

### Non-drawable matrices

**Figure 14:**
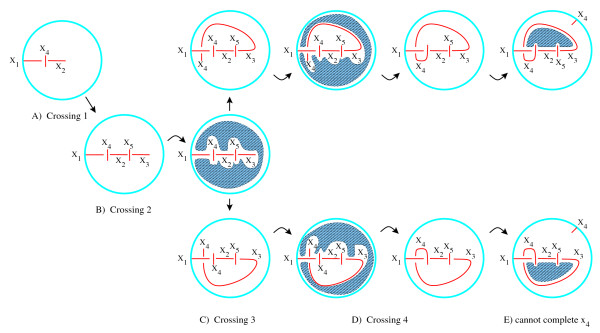
The matrix in Eqn. (4) does not correspond to a 3-string tangle.

Not all generated matrices correspond to a tangle. We use an algorithm almost identical to that described in [18] to completely determine if a matrix corresponds to a drawable tangle. This algorithm determines if all arcs can be drawn or if an arc becomes trapped in a region and cannot be completed. We illustrate with an example. If the matrix in Eqn. (4) corresponds to a coloring matrix of a tangle, then since it has five rows, the tangle must have five crossings. Also, based upon the pattern of 1's in this matrix, the first string should consist of four arcs, *x*_1_, *x*_2_, *x*_2_, *x*_4_, while the second string consists of arcs *x*_5_, *x*_6 _and the third string consists of arcs *x*_7 _and *x*_8_. A matrix corresponds to a tangle diagram if we can embed all of the arcs. In this case we say that the matrix is drawable. In order to determine if there exists a tangle diagram associated to the matrix in Eqn. (4), we begin by drawing the arcs *x*_1 _and *x*_2_. Recall the first row represents the first crossing with underarcs *x*_1_, *x*_2_. Since a -2 appears in the first row and the fourth column, we know that *x*_4 _crosses over between *x*_1 _and *x*_2_. Hence we also draw a portion of the arc *x*_4 _between the arcs *x*_1 _and *x*_2 _(Fig. 14A). Similarly since a -2 appears in the second row and fifth column, we know that *x*_5 _crosses over between *x*_2 _and *x*_3 _(Fig. 14B).

x1x2x3x4x5x6x7x8crossing 1crossing 2crossing 3crossing 4crossing 5 (110−200000110−2000−2011000000−201100−20000011)     (4)
 MathType@MTEF@5@5@+=feaafiart1ev1aaatCvAUfKttLearuWrP9MDH5MBPbIqV92AaeXatLxBI9gBaebbnrfifHhDYfgasaacH8akY=wiFfYdH8Gipec8Eeeu0xXdbba9frFj0=OqFfea0dXdd9vqai=hGuQ8kuc9pgc9s8qqaq=dirpe0xb9q8qiLsFr0=vr0=vr0dc8meaabaqaciaacaGaaeqabaqabeGadaaakqaabeqaauaabeqabKaaaaaabaaabaGamaiGitaaamiEaG3aiaiGitaaaSbaaSqaiaiGitaaaiadaciYeaaaigdaXaqajaiGitaaaaaakeaacWaGaIjbaaWG4baEdGaGaIjbaaWgaaWcbGaGaIjbaaGamaiGysaaaGOmaidabKaGaIjbaaaaaOqaaiadacixeaaadIha4nacacixeaaaBaaaleacacixeaaacWaGaYfbaaaIZaWmaeqcacixeaaaaaGcbaGamaiGysaaamiEaG3aiaiGysaaaSbaaSqaiaiGysaaaiadaciMeaaaisda0aqajaiGysaaaaaakeaacWaGacfbaaWG4baEdGaGacfbaaWgaaWcbGaGacfbaaGamaiGqraaaGynaudabKaGacfbaaaaaOqaaiadaci=daaadIha4nacaci=daaaBaaaleacaci=daaacWaGaY=aaaaI2aGnaeqcaci=daaaaaGcbaGamaiGeoaaamiEaG3aiaiGeoaaaSbaaSqaiaiGeoaaaiadaciHdaaaiEda3aqajaiGeoaaaaaakeaacWaGasVaaaWG4baEdGaGasVaaaWgaaWcbGaGasVaaaGamaiG0laaaGioaGdabKaGasVaaaaaaaaakeaafaqabeqbbaaaaeaacqWGJbWycqWGYbGCcqWGVbWBcqWGZbWCieGacqWFZbWCcqWFPbqAcqWFUbGBcqWGNbWzcqqGGaaicqaIXaqmaeaacqWGJbWycqWGYbGCcqWGVbWBcqWGZbWCcqWFZbWCcqWFPbqAcqWFUbGBcqWGNbWzcqqGGaaicqaIYaGmaeaacqWGJbWycqWGYbGCcqWGVbWBcqWGZbWCcqWFZbWCcqWFPbqAcqWFUbGBcqWGNbWzcqqGGaaicqaIZaWmaeaacqWGJbWycqWGYbGCcqWGVbWBcqWGZbWCcqWFZbWCcqWFPbqAcqWFUbGBcqWGNbWzcqqGGaaicqaI0aanaeaacqWGJbWycqWGYbGCcqWGVbWBcqWGZbWCcqWFZbWCcqWFPbqAcqWFUbGBcqWGNbWzcqqGGaaicqaI1aqnaaGaeeiiaaYaaeWaaeaafaqabeqbiauaaebabaGaeGymaedabaGaeGymaedabaGaeGimaadabaGaeyOeI0IaeGOmaidabaGaeGimaadabaGaeGimaadabaGaeGimaadabaGaeGimaadabaGaeGimaadabaGaeGymaedabaGaeGymaedabaGaeGimaadabaGaeyOeI0IaeGOmaidabaGaeGimaadabaGaeGimaadabaGaeGimaadabaGaeyOeI0IaeGOmaidabaGaeGimaadabaGaeGymaedabaGaeGymaedabaGaeGimaadabaGaeGimaadabaGaeGimaadabaGaeGimaadabaGaeGimaadabaGaeGimaadabaGaeyOeI0IaeGOmaidabaGaeGimaadabaGaeGymaedabaGaeGymaedabaGaeGimaadabaGaeGimaadabaGaeyOeI0IaeGOmaidabaGaeGimaadabaGaeGimaadabaGaeGimaadabaGaeGimaadabaGaeGimaadabaGaeGymaedabaGaeGymaedaaaGaayjkaiaawMcaaiaaxMaacaWLjaWaaeWaaeaacqaI0aanaiaawIcacaGLPaaaaaaa@F5DE@

In order to complete arc *x*_3_, we note that *x*_1 _crosses over between *x*_3 _and *x*_4 _(-2 appears in the third row and first column and hence the *x*_1 _is the overcrossing for this third crossing). Since *x*_1 _has already been drawn, we determine if the arc *x*_1 _is reachable from *x*_3 _by searching the region accessible to *x*_3 _(Fig. 14C, middle). In this case we see that *x*_3 _can reach *x*_1 _from both above and below and hence both cases are checked. Thus we draw the arc *x*_3 _approaching *x*_1 _from above in one case (Fig. 14C, top) and from below in the other case (Fig. 14C, bottom). We also draw the beginning part of the arc *x*_4_.

A portion of the arc *x*_4 _has been draw before (crossing over between *x*_1 _and *x*_2_), so we must determine if we can connect the previously drawn part of *x*_4 _with the beginning part of *x*_4 _that we just added. We determine if the previously drawn portion of *x*_4 _is within the region accessible to the newly drawn beginning part of *x*_4 _(Fig. 14D, left). Note that exactly one side of the previously drawn part of *x*_4 _is accessible. Hence there is exactly one way of connecting these two parts of *x*_4 _(Fig. 14D, right).

According to the matrix in Eqn. (4), the first string consists of exactly four arcs. Hence *x*_4 _must also connect to the boundary of the tangle ball. Therefore we check if the boundary of the tangle ball is accessible to the first part of *x*_4 _(Fig. 14E). It is not. After passing over between the arcs, *x*_1 _and *x*_2_, the arc *x*_4 _arc is trapped in the shaded region and cannot connect to the boundary of the 3-ball without introducing an extra crossing. Thus the matrix in Eqn. (4) does not correspond to a drawable tangle.

This is all done computationally. Currently no tangle diagrams are literally drawn. For a full description of the algorithm applied to link diagrams, see [18]. The main difference between our algorithm and the algorithm in [18] is that since we are interested in tangles, we must consider the boundary of the tangle 3-ball as shown in the example in Fig. 14.

### 2-string tangle simplification

**Figure 15:**
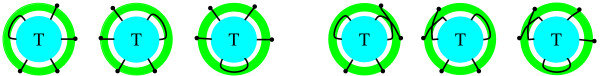
2-string tangle analysis will be used to determine these 2-string tangles from Fig. 6. The ends of the two strings are marked by dots.

Coloring is a weak knot invariant, but a strong tangle invariant. Hence, in order to use this invariant for solving tangle equations, we must first simplify the system of tangle equations in Fig. 6 by applying 2-string tangle analysis. Recall that the tangle **T **in Fig. 6 contains three strings. Observe that one of the strings in the green annulus loops back, connecting two of the three strings in the tangle **T **(see also Fig. 15 and the example in Fig. 5). Hence if we combine the three strings in the tangle **T **with the strings in the green annulus, we obtain a 2-string tangle. Thus the tangles in Fig. 15 are 2-string tangles. Endpoints of the two strings are marked by dots (note two strings have four endpoints).

We can solve for the 2-string tangles in Fig. 15 using the tangle equations in Fig. 6. This step requires some mathematical background in tangle analysis, although there is software (available at KnotPlot.com) for solving some 2-string tangle equations [38]. For information on how to solve 2-string tangle equations, see [1,33]. For additional 2-string tangle analysis applied to the Mu transpososome, see (Darcy IK, Luecke J, Vazquez M: A tangle analysis of the Mu transpososome protein complex which binds three DNA segments, manuscript in preparation).

**Figure 16:**
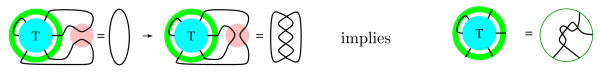
Solving for a 2-string tangle.

We can use a theorem in [29] and tangle calculus [1] (or tangle software [38]) to solve for one of these 2-string tangles (Fig. 16, where the crossings are either all right-handed or all left-handed).

**Figure 17:**
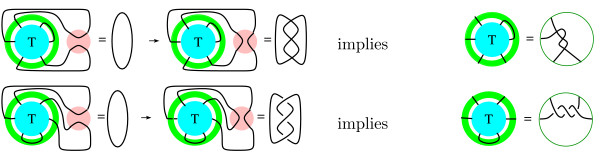
Solving for two more 2-string tangles.

Similarly, by [36] and tangle calculus [1] (or tangle software [38]), we can solve for two more of these 2-string tangles (Fig. 17, where the crossings are either all right-handed or all left-handed).

**Figure 18:**
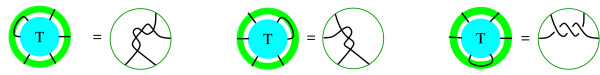
Tangle equations (crossings are either all right-handed or all left-handed). This system of tangle equations is equivalent to the system of tangle equation in Fig. 6 in that both systems have the same solution set.

This determines the remaining 2-string tangles in Fig. 15 since the last three tangles in Fig. 15 can be obtained from the first three by adding a crossing. In fact solving the system of tangle equations in Fig. 6 is equivalent to solving the system of three tangle equations in Fig. 18 for the 3-string tangle **T**. Observe, also, that the first 2-string tangle in Fig. 18 contains four right-handed or four left-handed crossings. Hence in order to obtain a five crossing knotted product, the extra crossing in the green annulus in the top right tangle equation in Fig. 6 must be of the same handedness as these four crossings. Thus the crossings in the five crossing knotted product must be either all right- or all left-handed.

### Checking the coloring invariants

**Figure 19:**
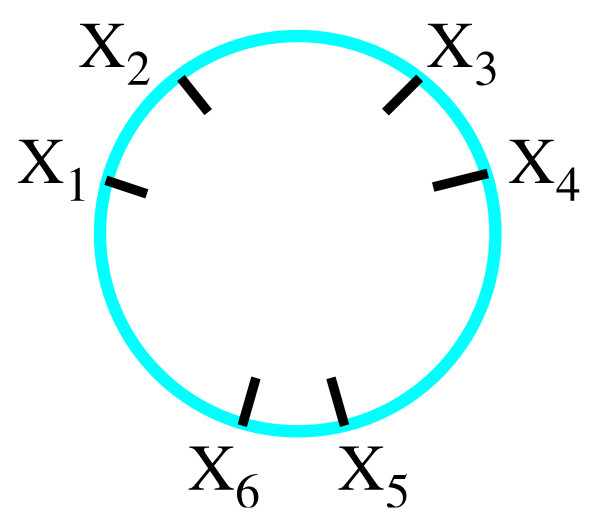
Re-labeled endpoint arcs. When the coloring invariants are determined, the columns corresponding to these endpoint arcs will be listed consecutively in the order shown and in the rightmost columns of the coloring matrix.

We first check if a generated matrix could be the coloring matrix of a 3-string tangle, **T**, which satisfies the system of tangle equations in Fig. 18. In order to use the coloring invariants, **M**_**l**_(**T**), **d**_**u**_(**T**), of this 3-string tangle, we must first move the six columns corresponding to the endpoint arcs so that they become the six rightmost columns of the coloring matrix. For convenience, we will re-label these endpoint arcs as *x*_1_, *x*_2 _,..., *x*_6 _as shown in Fig. 19.

Given a 3-string tangle **T **with *k *crossings, let **M**_**T **_be its *k *× (*k *+ 3) coloring matrix. Let *O*_*p *× (*k*-3) _be a *p *× (*k *- 3) matrix with all zero entries. Suppose for some (*k-*3) × (*k*-3) matrix *A*_(*k*-3) × (*k*-3)_, 3 × 6 matrix *M*_3 × 6 _in standard echelon form and some (*k*-3) × 6 matrix *B*_(*k*-3) × 6_, *SF*(**M_T_**) is as in Eqn. (5):

SF(MT)=(A(k−3)×(k−3)B(k−3)×603×(k−3)M3×6)     (5)
 MathType@MTEF@5@5@+=feaafiart1ev1aaatCvAUfKttLearuWrP9MDH5MBPbIqV92AaeXatLxBI9gBaebbnrfifHhDYfgasaacH8akY=wiFfYdH8Gipec8Eeeu0xXdbba9frFj0=OqFfea0dXdd9vqai=hGuQ8kuc9pgc9s8qqaq=dirpe0xb9q8qiLsFr0=vr0=vr0dc8meaabaqaciaacaGaaeqabaqabeGadaaakeaacqWGtbWucqWGgbGrcqGGOaakieqacqWFnbqtdaWgaaWcbaGae8hvaqfabeaakiabcMcaPiabg2da9maabmaabaqbaeqabiGaeqqaaiabdgeabnaaBaaaleaacqGGOaakcqWGRbWAcqGHsislcqaIZaWmcqGGPaqkcqGHxdaTcqGGOaakcqWGRbWAcqGHsislcqaIZaWmcqGGPaqkaeqaaaGcbaGaemOqai0aaSbaaSqaaiabcIcaOiabdUgaRjabgkHiTiabiodaZiabcMcaPiabgEna0kabiAda2aqabaaakeaacqaIWaamdaWgaaWcbaGaeG4mamJaey41aqRaeiikaGIaem4AaSMaeyOeI0IaeG4mamJaeiykaKcabeaaaOqaaiabd2eannaaBaaaleaacqaIZaWmcqGHxdaTcqaI2aGnaeqaaaaaaOGaayjkaiaawMcaaiaaxMaacaWLjaWaaeWaaeaacqaI1aqnaiaawIcacaGLPaaaaaa@5E9B@

**Figure 20:**
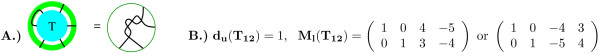
A.) The 2-string tangle **T**_**12**_. This 2-string tangle is obtained from the 3-string tangle, **T**, by connecting endpoint arcs *x*_1 _and *x*_2_. B.) The coloring invariants corresponding to the 2-string tangle **T**_**12**_.

If **T **is a solution to the system of tangle equations in Fig. 18, then connecting the endpoint arcs, *x*_1 _and *x*_2 _of **T **results in the four crossing 2-string tangle **T**_**12 **_shown in Fig. 20A. The coloring invariants of **T**_**12 **_are shown in Fig. 20B.

Connecting endpoint arcs *x*_1 _and *x*_2 _of **T **to obtain the 2-string tangle **T**_**12 **_results in adding the equation *x*_1 _- *x*_2_*= *0 to the matrix **M**_**T **_to obtain the matrix MT12
 MathType@MTEF@5@5@+=feaafiart1ev1aaatCvAUfKttLearuWrP9MDH5MBPbIqV92AaeXatLxBI9gBaebbnrfifHhDYfgasaacH8akY=wiFfYdH8Gipec8Eeeu0xXdbba9frFj0=OqFfea0dXdd9vqai=hGuQ8kuc9pgc9s8qqaq=dirpe0xb9q8qiLsFr0=vr0=vr0dc8meaabaqaciaacaGaaeqabaqabeGadaaakeaaieqacqWFnbqtdaWgaaWcbaGae8hvaq1aaSbaaWqaaiab=fdaXiab=jdaYaqabaaaleqaaaaa@3136@ (Eqn. 6).

MT12=(A(k−3)×(k−3)B(k−3)×603×(k−3)M3×601×(k−3)1−10000)     (6)
 MathType@MTEF@5@5@+=feaafiart1ev1aaatCvAUfKttLearuWrP9MDH5MBPbIqV92AaeXatLxBI9gBaebbnrfifHhDYfgasaacH8akY=wiFfYdH8Gipec8Eeeu0xXdbba9frFj0=OqFfea0dXdd9vqai=hGuQ8kuc9pgc9s8qqaq=dirpe0xb9q8qiLsFr0=vr0=vr0dc8meaabaqaciaacaGaaeqabaqabeGadaaakeaaieqacqWFnbqtdaWgaaWcbaGae8hvaq1aaSbaaWqaaiab=fdaXiab=jdaYaqabaaaleqaaOGaeyypa0ZaaeWaaeaafaqabeWacqbbbaGaemyqae0aaSbaaSqaaiabcIcaOiabdUgaRjabgkHiTiabiodaZiabcMcaPiabgEna0kabcIcaOiabdUgaRjabgkHiTiabiodaZiabcMcaPaqabaaakeaacqWGcbGqdaWgaaWcbaGaeiikaGIaem4AaSMaeyOeI0IaeG4mamJaeiykaKIaey41aqRaeGOnaydabeaaaOqaaiabicdaWmaaBaaaleaacqaIZaWmcqGHxdaTcqGGOaakcqWGRbWAcqGHsislcqaIZaWmcqGGPaqkaeqaaaGcbaGaemyta00aaSbaaSqaaiabiodaZiabgEna0kabiAda2aqabaaakeaacqaIWaamdaWgaaWcbaGaeGymaeJaey41aqRaeiikaGIaem4AaSMaeyOeI0IaeG4mamJaeiykaKcabeaaaOqaauaabeqabyaaaaqaaiabigdaXaqaaiabgkHiTiabigdaXaqaaiabicdaWaqaaiabicdaWaqaaiabicdaWaqaaiabicdaWaaaaaaacaGLOaGaayzkaaGaaCzcaiaaxMaadaqadaqaaiabiAda2aGaayjkaiaawMcaaaaa@6C79@

If **T **is a solution to the tangle equation in Fig. 20A, then this (*k *+ 1) × (*k *+ 3) matrix, MT12
 MathType@MTEF@5@5@+=feaafiart1ev1aaatCvAUfKttLearuWrP9MDH5MBPbIqV92AaeXatLxBI9gBaebbnrfifHhDYfgasaacH8akY=wiFfYdH8Gipec8Eeeu0xXdbba9frFj0=OqFfea0dXdd9vqai=hGuQ8kuc9pgc9s8qqaq=dirpe0xb9q8qiLsFr0=vr0=vr0dc8meaabaqaciaacaGaaeqabaqabeGadaaakeaaieqacqWFnbqtdaWgaaWcbaGae8hvaq1aaSbaaWqaaiab=fdaXiab=jdaYaqabaaaleqaaaaa@3136@, is a coloring matrix for **T**_**12**_. Since **d**_**u**_(**T**_**12**_) = 1, we know that the upper left (*k *+ 1 - 2) × (*k *+ 3 - 4) submatrix of the (*k *+ 1) × (*k *+ 3) matrix *SF*(MT12
 MathType@MTEF@5@5@+=feaafiart1ev1aaatCvAUfKttLearuWrP9MDH5MBPbIqV92AaeXatLxBI9gBaebbnrfifHhDYfgasaacH8akY=wiFfYdH8Gipec8Eeeu0xXdbba9frFj0=OqFfea0dXdd9vqai=hGuQ8kuc9pgc9s8qqaq=dirpe0xb9q8qiLsFr0=vr0=vr0dc8meaabaqaciaacaGaaeqabaqabeGadaaakeaaieqacqWFnbqtdaWgaaWcbaGae8hvaq1aaSbaaWqaaiab=fdaXiab=jdaYaqabaaaleqaaaaa@3136@) has determinant equal to 1. Since this matrix is in standard echelon form, this upper left (*k *- 1) × (*k *- 1) submatrix must be the identity matrix, *I*_(*k*-1) × (*k*-1)_, which has 1's along the diagonal and zero's elsewhere. Thus *A*_(*k*-3) × (*k*-3) _is the (*k *- 3) × (*k *- 3) identity matrix, *I*_(*k*-3) × (*k*-3)_. We also know that the lower right-hand corner 2 × 4 submatrix of *SF*(MT12
 MathType@MTEF@5@5@+=feaafiart1ev1aaatCvAUfKttLearuWrP9MDH5MBPbIqV92AaeXatLxBI9gBaebbnrfifHhDYfgasaacH8akY=wiFfYdH8Gipec8Eeeu0xXdbba9frFj0=OqFfea0dXdd9vqai=hGuQ8kuc9pgc9s8qqaq=dirpe0xb9q8qiLsFr0=vr0=vr0dc8meaabaqaciaacaGaaeqabaqabeGadaaakeaaieqacqWFnbqtdaWgaaWcbaGae8hvaq1aaSbaaWqaaiab=fdaXiab=jdaYaqabaaaleqaaaaa@3136@) is equal to **M**_**l**_(**T**_**12**_). Thus if **T **is a solution to the tangle equation in Fig. 20A, *SF*(MT12
 MathType@MTEF@5@5@+=feaafiart1ev1aaatCvAUfKttLearuWrP9MDH5MBPbIqV92AaeXatLxBI9gBaebbnrfifHhDYfgasaacH8akY=wiFfYdH8Gipec8Eeeu0xXdbba9frFj0=OqFfea0dXdd9vqai=hGuQ8kuc9pgc9s8qqaq=dirpe0xb9q8qiLsFr0=vr0=vr0dc8meaabaqaciaacaGaaeqabaqabeGadaaakeaaieqacqWFnbqtdaWgaaWcbaGae8hvaq1aaSbaaWqaaiab=fdaXiab=jdaYaqabaaaleqaaaaa@3136@) is as in Eqn. 7 where * represents an arbitrary integer.

SF(M12)=(I(k−3)×(k−3)B(k−3)×604×(k−3)1000**0100**00104−500013−4) or (I(k−3)×(k−3)B(k−3)×604×(k−3)1000**0100**0010−430001−54)     (7)
 MathType@MTEF@5@5@+=feaafiart1ev1aaatCvAUfKttLearuWrP9MDH5MBPbIqV92AaeXatLxBI9gBaebbnrfifHhDYfgasaacH8akY=wiFfYdH8Gipec8Eeeu0xXdbba9frFj0=OqFfea0dXdd9vqai=hGuQ8kuc9pgc9s8qqaq=dirpe0xb9q8qiLsFr0=vr0=vr0dc8meaabaqaciaacaGaaeqabaqabeGadaaakeaacqWGtbWucqWGgbGrcqGGOaakieqacqWFnbqtdaWgaaWcbaGae8xmaeJae8NmaidabeaakiabcMcaPiabg2da9maabmaabaqbaeqabiGaeqqaaiabdMeajnaaBaaaleaacqGGOaakcqWGRbWAcqGHsislcqaIZaWmcqGGPaqkcqGHxdaTcqGGOaakcqWGRbWAcqGHsislcqaIZaWmcqGGPaqkaeqaaaGcbaGaemOqai0aaSbaaSqaaiabcIcaOiabdUgaRjabgkHiTiabiodaZiabcMcaPiabgEna0kabiAda2aqabaaakeaacqaIWaamdaWgaaWcbaGaeGinaqJaey41aqRaeiikaGIaem4AaSMaeyOeI0IaeG4mamJaeiykaKcabeaaaOqaauaabeqaeyaeaababaGaeGymaedabaGaeGimaadabaGaeGimaadabaGaeGimaadabaGaeiOkaOcabaGaeiOkaOcabaGaeGimaadabaGaeGymaedabaGaeGimaadabaGaeGimaadabaGaeiOkaOcabaGaeiOkaOcabaGaeGimaadabaGaeGimaadabaGaeGymaedabaGaeGimaadabaGaeGinaqdabaGaeyOeI0IaeGynaudabaGaeGimaadabaGaeGimaadabaGaeGimaadabaGaeGymaedabaGaeG4mamdabaGaeyOeI0IaeGinaqdaaaaaaiaawIcacaGLPaaacqqGGaaicqqGVbWBcqqGYbGCcqqGGaaidaqadaqaauaabeqaciabeeaacqWGjbqsdaWgaaWcbaGaeiikaGIaem4AaSMaeyOeI0IaeG4mamJaeiykaKIaey41aqRaeiikaGIaem4AaSMaeyOeI0IaeG4mamJaeiykaKcabeaaaOqaaiabdkeacnaaBaaaleaacqGGOaakcqWGRbWAcqGHsislcqaIZaWmcqGGPaqkcqGHxdaTcqaI2aGnaeqaaaGcbaGaeGimaaZaaSbaaSqaaiabisda0iabgEna0kabcIcaOiabdUgaRjabgkHiTiabiodaZiabcMcaPaqabaaakeaafaqabeabgabaqaqaaiabigdaXaqaaiabicdaWaqaaiabicdaWaqaaiabicdaWaqaaiabcQcaQaqaaiabcQcaQaqaaiabicdaWaqaaiabigdaXaqaaiabicdaWaqaaiabicdaWaqaaiabcQcaQaqaaiabcQcaQaqaaiabicdaWaqaaiabicdaWaqaaiabigdaXaqaaiabicdaWaqaaiabgkHiTiabisda0aqaaiabiodaZaqaaiabicdaWaqaaiabicdaWaqaaiabicdaWaqaaiabigdaXaqaaiabgkHiTiabiwda1aqaaiabisda0aaaaaaacaGLOaGaayzkaaGaaCzcaiaaxMaadaqadaqaaiabiEda3aGaayjkaiaawMcaaaaa@B052@

Hence, in order to determine if a matrix could correspond to a tangle, **T**, which is a solution to the tangle equation in Fig. 20A, we check if MT12
 MathType@MTEF@5@5@+=feaafiart1ev1aaatCvAUfKttLearuWrP9MDH5MBPbIqV92AaeXatLxBI9gBaebbnrfifHhDYfgasaacH8akY=wiFfYdH8Gipec8Eeeu0xXdbba9frFj0=OqFfea0dXdd9vqai=hGuQ8kuc9pgc9s8qqaq=dirpe0xb9q8qiLsFr0=vr0=vr0dc8meaabaqaciaacaGaaeqabaqabeGadaaakeaaieqacqWFnbqtdaWgaaWcbaGae8hvaq1aaSbaaWqaaiab=fdaXiab=jdaYaqabaaaleqaaaaa@3136@ is row equivalent to one of the two matrices in Eqn. (7). This is not a guarantee that **T **is a solution as different tangles can have the same coloring invariants [15], but our computational results show that it is sufficient for solving the tangle equations in Fig. 18.

**Figure 21:**
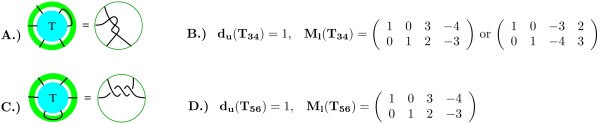
A.) The 2-string tangle **T**_**34**_. This 2-string tangle is obtained from the 3-string tangle, **T**, by connecting endpoint arcs *x*_3 _and *x*_4_. B.) The coloring invariants corresponding to the 2-string tangle **T**_**34**_. C.) The 2-string tangle **T**_**56**_. This 2-string tangle is obtained from the 3-string tangle, **T**, by connecting endpoint arcs *x*_5 _and *x*_6_. D.) The coloring invariants corresponding to the 2-string tangle **T**_**56**_.

Similarly to determine if **T **could be a solution to the tangle equation in Fig. 21A, we add the equation *x*_3 _- *x*_4 _= 0 to the matrix **M**_**T **_and check if this matrix satisfies the coloring invariants of **T**_**34 **_as shown in Fig 21B. Finally, we determine if **T **could be a solution to the tangle equation in Fig. 21C, by adding the equation *x*_5 _- *x*_6 _= 0 to the matrix **M**_**T **_and checking if this matrix satisfies the coloring invariants of **T**_**56 **_as shown in Fig. 21D.

Alternatively, we can determine what the entries of the submatrix *M*_3 × 6 _of **M**_**T **_(Eqn. (5)) need to be in order for **T **to satisfy the tangle equations in Fig. 18. To determine *M*_3 × 6_, we add the equations *x*_*i *_- *x*_(i+1)_ for each *i *= 1, 3, 5, and determine the constraints needed to satisfy the coloring invariants of **T**_**i**(**i+1**)_. If **T **satisfies the tangle equations in Fig. 18, then the determinant of *A*, the upper left (*k *- 3) × (*k *- 3) submatrix of **M**_**T**_, is 1 and *M*_3 × 6 _is as in Eqn. (8).

M3×6∼(1−11−11−101t−1−ts−r−x−s+r+x00x1−xr+x−1−r−x)     (8)
 MathType@MTEF@5@5@+=feaafiart1ev1aaatCvAUfKttLearuWrP9MDH5MBPbIqV92AaeXatLxBI9gBamXvP5wqSXMqHnxAJn0BKvguHDwzZbqegyvzYrwyUfgarqqtubsr4rNCHbGeaGqiA8vkIkVAFgIELiFeLkFeLk=iY=Hhbbf9v8qqaqFr0xc9pk0xbba9q8WqFfeaY=biLkVcLq=JHqVepeea0=as0db9vqpepesP0xe9Fve9Fve9GapdbaqaaeGacaGaaiaabeqaamqadiabaaGcbaGaemyta00aaSbaaSqaaiabiodaZiabgEna0kabiAda2aqabaGccqWI8iIodaqadaqaauaabeqadyaaaaqaaiabigdaXaqaaiabgkHiTiabigdaXaqaaiabigdaXaqaaiabgkHiTiabigdaXaqaaiabigdaXaqaaiabgkHiTiabigdaXaqaaiabicdaWaqaaiabigdaXaqaaiabdsha0bqaaiabgkHiTiabigdaXiabgkHiTiabdsha0bqaaiabdohaZjabgkHiTiabdkhaYjabgkHiTiabdIha4bqaaiabgkHiTiabdohaZjabgUcaRiabdkhaYjabgUcaRiabdIha4bqaaiabicdaWaqaaiabicdaWaqaaiabdIha4bqaaiabigdaXiabgkHiTiabdIha4bqaaiabigdaXiabgUcaRiabdIha4bqaaiabgkHiTiabigdaXiabgkHiTiabdkhaYjabgkHiTiabdIha4baaaiaawIcacaGLPaaacaWLjaGaaCzcamaabmaabaGaeGioaGdacaGLOaGaayzkaaaaaa@76A2@

for some integer *x*, where *r *= 3 or -5, *s *= 2 or -4, and *t *= 2. As a check, both methods were implemented.

### Equivalence moves

Recall that a tangle can be represented by a number of different diagrams related by Reidemeister moves. While generating matrices, we omit matrices where the corresponding diagram can be simplified by RI or RII moves and other matrix related moves (as described in the subsection **Tangle generation**). We also added two additional equivalence relations.

**Figure 22:**
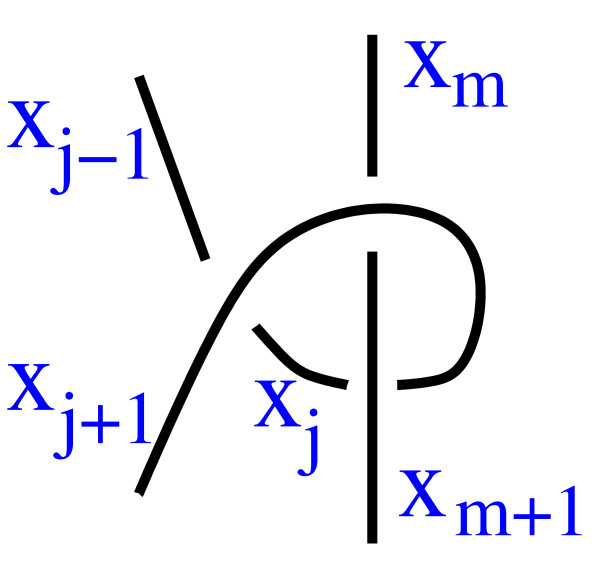
A diagram corresponding to Eqn. 9.

We removed tangles containing the diagram shown in Fig. 22 by removing matrices containing the submatrices in Eqn. (9).

(xj−1xjxj+1xmxm±1i11−200i+10110−2k00−211)&(xj−1xjxj+1xmxm±1i−11100−2i−21100k−20011)     (9)
 MathType@MTEF@5@5@+=feaafiart1ev1aaatCvAUfKttLearuWrP9MDH5MBPbIqV92AaeXatLxBI9gBaebbnrfifHhDYfgasaacH8akY=wiFfYdH8Gipec8Eeeu0xXdbba9frFj0=OqFfea0dXdd9vqai=hGuQ8kuc9pgc9s8qqaq=dirpe0xb9q8qiLsFr0=vr0=vr0dc8meaabaqaciaacaGaaeqabaqabeGadaaakeaadaqadaqaauaabeqaeyaraqqabaaabaGaemiEaG3aaSbaaSqaaiabdQgaQjabgkHiTiabigdaXaqabaaakeaacqWG4baEdaWgaaWcbaGaemOAaOgabeaaaOqaaiabdIha4naaBaaaleaacqWGQbGAcqGHRaWkcqaIXaqmaeqaaaGcbaGaemiEaG3aaSbaaSqaaiabd2gaTbqabaaakeaacqWG4baEdaWgaaWcbaGaemyBa0MaeyySaeRaeGymaedabeaaaOqaaiabdMgaPbqaaiabigdaXaqaaiabigdaXaqaaiabgkHiTiabikdaYaqaaiabicdaWaqaaiabicdaWaqaaiabdMgaPjabgUcaRiabigdaXaqaaiabicdaWaqaaiabigdaXaqaaiabigdaXaqaaiabicdaWaqaaiabgkHiTiabikdaYaqaaiabdUgaRbqaaiabicdaWaqaaiabicdaWaqaaiabgkHiTiabikdaYaqaaiabigdaXaqaaiabigdaXaaaaiaawIcacaGLPaaacqGGMaGjdaqadaqaauaabeqaeyaraqqabaaabaGaemiEaG3aaSbaaSqaaiabdQgaQjabgkHiTiabigdaXaqabaaakeaacqWG4baEdaWgaaWcbaGaemOAaOgabeaaaOqaaiabdIha4naaBaaaleaacqWGQbGAcqGHRaWkcqaIXaqmaeqaaaGcbaGaemiEaG3aaSbaaSqaaiabd2gaTbqabaaakeaacqWG4baEdaWgaaWcbaGaemyBa0MaeyySaeRaeGymaedabeaaaOqaaiabdMgaPjabgkHiTiabigdaXaqaaiabigdaXaqaaiabigdaXaqaaiabicdaWaqaaiabicdaWaqaaiabgkHiTiabikdaYaqaaiabdMgaPbqaaiabgkHiTiabikdaYaqaaiabigdaXaqaaiabigdaXaqaaiabicdaWaqaaiabicdaWaqaaiabdUgaRbqaaiabgkHiTiabikdaYaqaaiabicdaWaqaaiabicdaWaqaaiabigdaXaqaaiabigdaXaaaaiaawIcacaGLPaaacaWLjaGaaCzcamaabmaabaGaeGyoaKdacaGLOaGaayzkaaaaaa@8E6A@

This also eliminates other tangle diagrams whose matrices contain these submatrices, but all such tangles can be simplified.

A tangle diagram containing the left-hand side of an RIII move (Fig. 8) will be equivalent to the tangle diagram obtained after the RIII move has been performed. Hence we choose one of these tangle diagrams and discard the other. After the above equivalence moves, we are left with thirteen possible tangles which can be checked by hand to determine if they correspond to equivalent or non-equivalent solutions to the tangle equations in Fig. 18 (or equivalently, Fig. 6).

## Authors' contributions

CM and JN contributed to the mathematical analysis for applying the coloring invariant. JN also drafted significant portions of the sections **The coloring invariants, Tangle generation, Checking the coloring invariants **. AP, JS, and TT developed the software implementing the coloring invariant calculations. RM contributed to the **Non-drawable **section and is responsible for the subroutine which determines if a matrix corresponds to a drawable tangle. He was assisted by ND and JS. JC, ND, SM, and JS developed equivalence moves which were implemented by ND, RM, and JS. ID conceived of and oversaw this project, drafted much of the manuscript, and contributed to the mathematical and software development. All authors read and approved the final manuscript.
